# Parasitological study of animals maintained under human care at a Zoobotanical Park in Bahia, Northeastern Brazil

**DOI:** 10.3389/fpara.2026.1929185

**Published:** 2026-10-15

**Authors:** Aksa Ingrid Vieira Batista, João Vitor de Oliveira Gurgel, Lucas Micael Freire Pereira, Paulo Roberto Bahiano Ferreira, Marta Calasans Gomes, Arthur Willian de Lima Brasil, Victor Fernando Santana Lima, Wesley Adson Costa Coelho, Lucas Rannier Ribeiro Antonino Carvalho, Josivania Soares Pereira

**Affiliations:** 1 Graduate Program in Animal Science, Universidade Federal da Paraíba, Areia, Paraíba, Brazil; 2 Universidade Federal Rural do Semi-Árido (UFERSA), Mossoró, Rio Grande do Norte, Brazil; 3 Universidade de Brasília (UnB), Plano Piloto, Brasília, Distrito Federal, Brazil; 4 Centro de Triagem de Animais Silvestres Oeste Bahia (CETAS Oeste), Instituto do Meio Ambiente e Recursos Hídricos (INEMA), Barreiras, Bahia, Brazil; 5 Centro de Triagem de Animais Silvestres na Bahia (CETAS-BA), Instituto do Meio Ambiente e Recursos Hídricos (INEMA), Cruz das Almas, Bahia, Brazil; 6 Universidade Federal da Paraíba (UFPB), João Pessoa, Paraíba, Brazil; 7 Universidade Federal de Sergipe (UFS Sertão), Nossa Sra. da Glória, Sergipe, Brazil; 8 Faculdade de Enfermagem e de Medicina Nova Esperança (FACENE), Mossoró, Rio Grande do Norte, Brazil; 9 Department of Physiology & Pharmacology, Karolinska Institutet, Stockholm, Sweden

**Keywords:** Cestoda, coccidia, coproparasitology, Nematoda, zoonoses

## Abstract

**Introduction:**

Coproparasitological analysis in zoo animals is essential for preventing parasitic diseases, which can compromise conservation efforts and pose occupational health risks. This study aimed to characterize the endoparasitic fauna detected in fecal samples from mammals, birds, and reptiles maintained at the Getúlio Vargas Zoobotanical Park, Salvador, Bahia, Brazil.

**Methods:**

Fecal samples were examined using four complementary coproparasitological methods: the direct method, spontaneous sedimentation, Willis flotation, and Mini-FLOTAC®.

**Results:**

Of the samples (N = 144), 52.8% were positive. Among these, 80.3% harbored nematodes, 23.7% protozoa, 13.2% cestodes, and 10.5% trematodes, with 26.3% presenting mixed infections. In primates, coccidia, *Strongyloides* spp., *Bertiella* spp., and *Platynosomum* spp. were observed; in carnivores, *Strongyloides* spp. and Oxyuridae; in Artiodactyla, *Balantioides* spp., *Trichuris* spp., and *Strongyloides* spp.; in *Perissodactyla, Balantioides* spp. and Strongyloidea; and in Didelphimorphia, Eimeria spp., *Cruzia* spp., and *Aspidodera* spp. In birds, *Capillaria* spp., *Isospora* spp., and cestodes were identified; in reptiles, *Nyctotherus* spp. and *Balantioides* spp. were detected.

**Discussion:**

This study reports, for the first time, the occurrence of *Strongyloides* spp. in *Ateles marginatus* and *Leontopithecus chrysomelas, Bertiella* spp. in *Callicebus barbarabrownae* and *Saimiri sciureus*, and *Capillaria* spp. in *Amazona rhodocorytha, Eupsittula aurea*, and *Diopsittca nobilis*.

## 1 Introduction

Brazil harbors approximately 20% of the world’s biodiversity, distributed across biomes such as the Caatinga, Atlantic Forest, Amazon, Pampa, Cerrado, and Pantanal (ICMBio, 2017; Batista et al., 2021; Prazeres Júnior et al., 2024; Pinto et al., 2026). This high biodiversity implies great responsibility in terms of conservation. Among ecological interactions, parasitism stands out due to its widespread occurrence, involving a large proportion of animal taxa and playing an important role in population regulation (Silva et al., 2022, 2025; Pitombeira et al., 2025).

Parasites can cause conditions ranging from subclinical infections to clinical alterations such as anorexia, weight loss, gastrointestinal disorders, anemia, and reduced survival and reproductive success (Reed et al., 2012; Camejo and Loughlin, 2015; Prazeres Júnior et al., 2024). In captive environments, such as zoos, these infections are frequent even in the presence of sanitary measures and may compromise conservation programs (Miranda et al., 2023).

The high occurrence of parasites in captivity is associated with limited space, increased host density, proximity among species, and stress conditions that may reduce immune response. Additionally, contact with synanthropic fauna facilitates the transmission of parasitic agents, making these infections difficult to control. This parasitoses impact animal welfare and may have zoonotic potential, representing an occupational risk (Schieber and Štrkolcová, 2019; Dashe and Berhanu, 2020; Patra et al., 2020; Batista et al., 2021).

The identification of parasites is essential to understand host–parasite interactions and to support management, control, and treatment strategies, as well as to contribute to the prevention of zoonoses (Batista et al., 2021). In this context, coproparasitological analysis stands out as a non-invasive and widely used method for the diagnosis of endoparasites. Among the available techniques, Willis flotation, spontaneous sedimentation, direct method, and Mini-FLOTAC^®^ are commonly used (Hoffmann et al., 1934; Willis-Mollay, 1921; Cringoli et al., 2010; 2013; 2017).

Despite their importance, coproparasitological studies in zoological settings remain scarce in Brazil and are generally restricted to specific animal groups. Therefore, this study aimed to characterize the endoparasitic fauna detected in fecal samples from different taxonomic groups maintained at the Getúlio Vargas Zoobotanical Park, Salvador, Bahia, using complementary coproparasitological methods, and to describe the distribution of the identified parasitic taxa among the evaluated hosts.

## 2 Methods

### 2.1 Study area

The Getúlio Vargas Zoobotanical Park covers an area of approximately 25 hectares, with remnants of secondary Atlantic Forest. The climate is classified as tropical hot and humid, with an average annual rainfall of approximately 1,800 mm and a mean annual temperature of 25 °C (INMET, 2022) (**Figure 1**).

**Figure 1:**
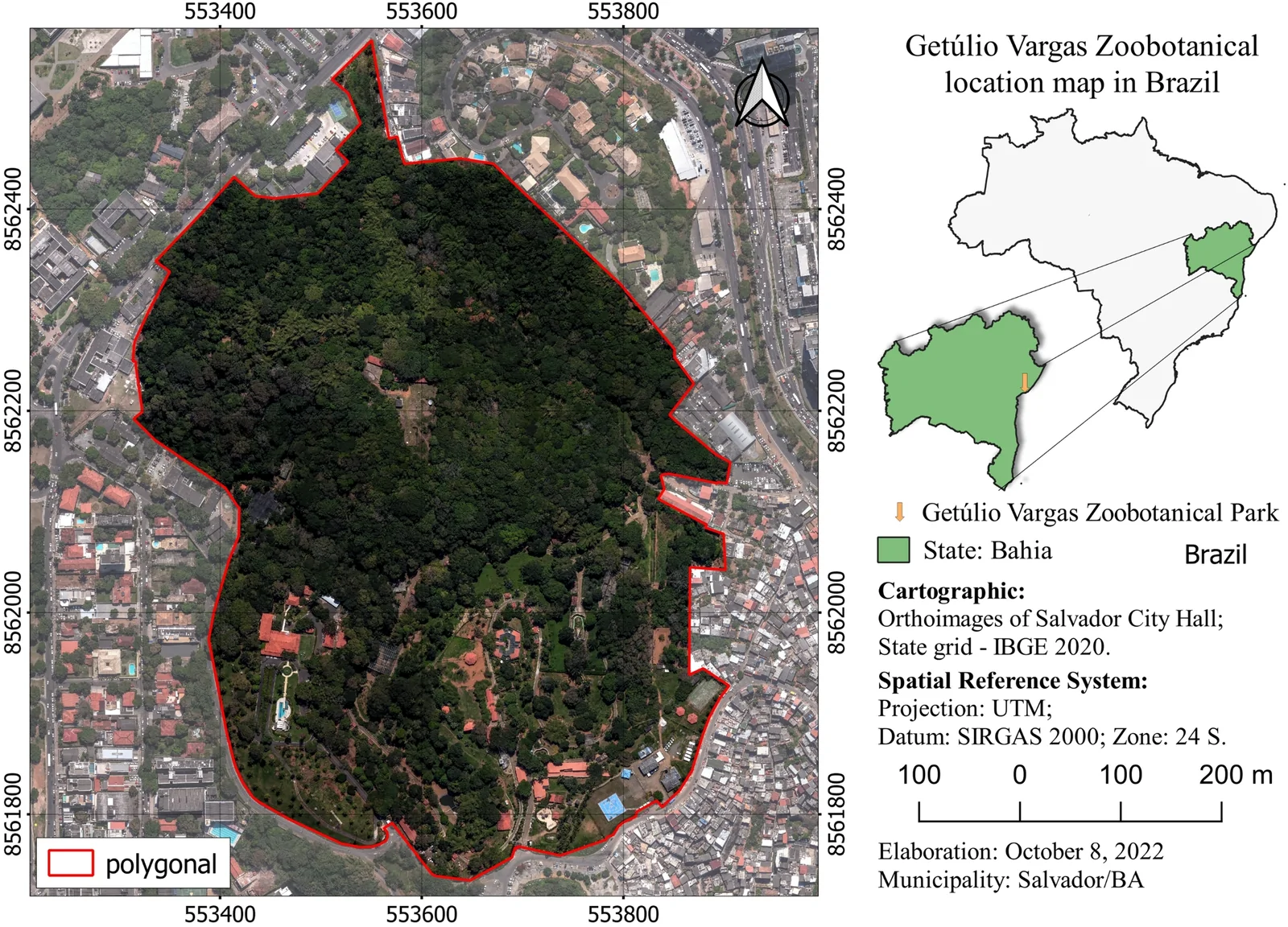
Location map of the Getúlio Vargas Zoobotanical Park (PZGV) in the city of Salvador, Bahia. Map lines delineate study areas and do not necessarily depict accepted national boundaries.

### 2.2 Animals used in the study

A total of 75 species and 409 individual animals were included, comprising 40 species (n=229) of mammals, 27 species (n=153) of birds, and seven species (n=27) of reptiles (**Table 1**). Sample collection was not possible for some individuals in the collection, particularly reptiles that did not defecate during the sampling period and those that spend extended periods in water, such as freshwater turtles and caimans. All procedures adopted in this study followed the guidelines established by the Ethics Committee on the Use of Animals in Research of the Universidade Federal Rural do Semi-Árido (CEUA-UFERSA), under protocol number 37/2020. The project was also submitted to and approved by the Biodiversity Authorization and Information System (SISBIO), under approval protocol number 77167-1.

**Table 1:** Summary of the animals included in the study, including the number of animals (NA) and samples (NS).

Class Mammalia				
Taxa	Common name	Scientific name	NA	NS
Order Carnivora				
Family Felidae	Jaguar	*Panthera onca*	5	5
	Cougar	*Puma concolor*	2	2
	Jaguarundi	*Puma yagouaroundi*	1	1
	Ocelot	*Leopardus pardalis*	2	2
	Margay	*Leopardus wiedii*	3	3
	Oncilla	*Leopardus tigrinus*	1	1
Family Procyonidae	Crab-eating raccoon	*Procyon cancrivorus*	3	2
Family Mustelidae	Tayra	*Eira barbara*	2	2
	Lesser grison	*Galictis cuja*	1	1
	Neotropical otter	*Lontra longicaudis*	1	1
Family Canidae	Maned wolf	*Chrysocyon brachyurus*	3	3
	Crab-eating fox	*Cerdocyon thous*	14	8
Family Ursidae	Andean bear	*Tremarctos ornatus*	2	2
Order Primates				
Family Atelidae	Black howler monkey	*Alouatta caraya*	7	4
	Black-faced spider monkey	*Ateles paniscus*	4	1
	White-cheeked spider monkey	*Ateles marginatus*	3	1
Family Cebidae	Yellow-breasted capuchin	*Sapajus xanthosternos*	47	6
	Blond capuchin	*Sapajus flavius*	20	3
	Crested capuchin	*Sapajus robustus*	1	1
	Common squirrel monkey	*Saimiri sciureus*	16	4
	Golden-headed lion tamarin	*Leontopithecus chrysomelas*	13	8
	Common marmoset	*Callithrix jacchus*	6	3
	Black-tufted marmoset	*Callithrix penicillata*	1	1
Family Pitheciidae	Blond titi monkey	*Callicebus barbarabrownae*	1	1
Family Aotidae	Night monkey	*Aotus trivirgatus*	2	2
Order Rodentia				
Family Erethizontidae	Brazilian porcupine	*Coendou prehensilis*	1	1
Family Hydrochoeridae	Capybara	*Hydrochoerus hydrochaeris*	1	1
Order Didelphimorphia				
Family Didelphidae	White-eared opossum	*Didelphis albiventris*	10	4
	Black-eared opossum	*Didelphis aurita*	8	2
Order Artiodactyla				
Family Cervidae	Gray brocket deer	*Subulo gouazoubira*	15	3
	Sambar deer	*Rusa unicolor*	17	2
	Marsh deer	*Blastocerus dichotomus*	1	1
Family Suidae	White-lipped peccary	*Tayassu pecari*	1	1
	Collared peccary	*Pecari tajacu*	3	1
Family Camelidae	Bactrian camel	*Camelus bactrianus*	1	1
Order Perissodactyla				
Family Equidae	Plains zebra	*Equus quagga burchelli*	2	2
Family Tapiridae	South American tapir	*Tapirus terrestris*	1	1
Order Pilosa				
Family Myrmecophagidae	Giant anteater	*Myrmecophaga tridactyla*	2	2
	Southern tamandua	*Tamandua tetradactyla*	4	1
Family Bradypodidae	Brown-throated sloth	*Bradypus variegatus*	1	1

Class Aves				
Order Psittaciformes				
Family Psittacidae	Macaws	*Ara spp.*	10	2
	Hyacinth macaw	*Anodorhynchus hyacinthinus*	12	1
	Blue-fronted amazon	*Amazona aestiva*	10	5
	Red-browed amazon	*Amazona rhodocorytha*	4	2
	Golden conure	*Guaruba guarouba*	3	2
	Peach-fronted parakeet	*Eupsittula aurea*	1	1
	Golden-capped parakeet	*Aratinga auricapillus*	1	1
	Red-shouldered macaw	*Diopsittaca nobilis*	30	1
	Red-fan parrot	*Deroptyus accipitrinus*	1	1
Order Passeriformes				
Family Turdidae	Rufous-bellied thrush	*Turdus rufiventris*	7	3
Family Thraupidae	Sayaca tanager	*Thraupis sayaca*	4	2
Family Hirundinidae	Blue-and-white swallow	*Pygochelidon cyanoleuca*	1	1
Family Corvidae	Curl-crested jay	*Cyanocorax cristatellus*	1	1
Family Icteridae	Chopi blackbird	*Gnorimopsar chopi*	1	1
Order Falconiformes				
Family Falconidae	Southern crested caracara	*Caracara plancus*	6	2
Order Accipitriformes				
Family Accipitridae	Roadside hawk	*Rupornis magnirostris*	3	1
	Black-chested buzzard-eagle	*Geranoaetus melanoleucus*	1	1
	Harpy eagle	*Harpia harpyja*	1	1
Order Pelecaniformes				
Family Ardeidae	Striated heron	*Butorides striata*	4	2
	Black-crowned night heron	*Nycticorax nycticorax*	2	1
Family Threskiornithidae	Scarlet ibis	*Eudocimus ruber*	40	1
Order Phoenicopteriformes				
Family Phoenicopteridae	Chilean flamingo	*Phoenicopterus chilensis*	4	1
Order Galliformes				
Family Phasianidae	Indian peafowl	*Pavo cristatus*	1	1
Family Cracidae	Bare-faced curassow	*Crax fasciolata*	1	1
Order Cariamiformes				
Family Cariamidae	Red-legged seriema	*Cariama cristata*	1	1
Order Piciformes				
Family Rhamphastidae	Toco toucan	*Rhamphastos toco*	2	2
Order Columbiformes				
Family Columbidae	Ground doves	*Columbina* spp.	1	1

Class Lepidosauria				
Order Squamata				
Family Boidae	Rainbow boa	*Epicrates* spp.	3	3
Family Pythonidae	Burmese python	*Python molurus*	6	2
Family Viperidae	Bushmaster	*Lachesis muta*	1	1
Family Colubridae	Corn snake	*Pantherophis guttatus*	10	1
Family Iguanidae	Green iguana	*Iguana iguana*	1	1

Class Archelosauria				
Order Testudines				
Family Testudinidae	Red-footed tortoise	*Chelonoidis carbonaria*	5	3
Order Crocodylia				
Family Alligatoridae	Broad-snouted caiman	*Caiman latirostris*	1	1

### 2.3 Biological sample collection

Fecal samples were collected in December 2021 as pooled samples directly from the ground of the enclosures, preferably during cleaning periods, in order to reduce animal stress and ensure handler safety. The study followed a cross-sectional design, with all samples collected during a single sampling campaign. Because fecal samples were collected as composite samples from enclosure floors, particularly in enclosures housing more than one animal, the enclosure was considered the sampling unit for all analysis. Consequently, statistical analyses were performed at the enclosure level rather than at the individual animal level. Although this method presents a risk of environmental contamination, measures were taken to minimize it, such as collecting inner portions of the fecal mass to avoid areas in contact with the substrate (soil). The samples were preserved in 5% formalin (1:2) and transported to the Animal Parasitology Laboratory at UFERSA for analysis.

### 2.4 Coproparasitological techniques

Sample processing was carried out from January to July 2022. Each fecal sample was subjected to direct method (Hoffmann, 1987), simple sedimentation (Hoffmann et al., 1934), flotation in a hypersaturated sodium chloride solution (Willis-Mollay, 1921), and flotation using the Mini-FLOTAC^®^ method (Cringoli et al., 2010; 2013; 2017). For the first three techniques, two slides were prepared per sample, each examined by two different evaluators in order to increase result reliability and reduce potential errors in microscopic reading.

#### 2.4.1 Direct method

The direct method proposed by Hoffmann (1987) was adapted, considering that, depending on fecal characteristics, microscopic examination could be hindered by intense coloration and the presence of food debris, hair, and other artifacts. Thus, approximately two grams of feces were placed in a plastic container, diluted in distilled water, and, when necessary, sieved to remove particles that could interfere with visualization. Lugol’s iodine was not used, as its staining may hinder the identification of eggs and oocysts, compromising the quality of diagnosis and photographic records. The slides were examined under light microscopy.

#### 2.4.2 Spontaneous sedimentation

The spontaneous sedimentation technique was performed by homogenizing approximately five grams of feces in 100 mL of distilled water. The suspension was then transferred to a sedimentation cup, and, after the decantation period, three successive washings were carried out, with the supernatant discarded and the sediment preserved. Using a plastic Pasteur pipette, one drop of the sediment was placed on a slide. Although the original technique recommends the addition of 1% Lugol’s iodine, it was not used in the present study to allow better visualization of parasitic structures. The slide was then covered with a coverslip and examined under light microscopy (Hoffmann et al., 1934).

#### 2.4.3 Willis flotation

The flotation technique using a hyper saturated sodium chloride solution was performed by diluting approximately two grams of feces in 20 mL of the solution, followed by homogenization. The material was then transferred to a test tube until a convex meniscus was formed, onto which a glass coverslip was placed and maintained in contact for approximately 15 minutes. After this period, the coverslip was carefully removed and placed onto a glass slide for examination under light microscopy (Willis-Mollay, 1921).

#### 2.4.4 Mini-FLOTAC^®^

In the Mini-FLOTAC^®^ technique, two grams of previously homogenized feces preserved in 5% formalin were used, to which 38 mL of hyper saturated sodium chloride solution were added, according to the dilution recommended by the manufacturer for dogs and cats. The suspension was homogenized again, sieved, and transferred to the Mini-FLOTAC^®^ chamber. After filling, a 10-minute period was allowed before rotating the reading disk and proceeding with observation under light microscopy (Cringoli et al., 2010; 2013; 2017; Capasso et al., 2019). The values of eggs per gram of feces (EPG), oocysts per gram of feces (OPG), and larvae per gram of feces (LPG) were obtained by multiplying the number of observed parasitic structures by 10. This method enables both qualitative and quantitative analyses, allowing the estimation of infection intensity (Cringoli et al., 2010; 2013; 2017; Capasso et al., 2019).

### 2.5 Parasite identification

Parasite identification was performed by experienced parasitologists using light microscopy based on the morphological characteristics of eggs, cysts, oocysts, larvae, and trophozoites, considering features such as size, shape, shell thickness, color, internal structures, and the presence of specific diagnostic characteristics. Taxonomic identification was based on standard parasitological identification keys and reference atlases (Bowman, 2021; Zajac and Conboy, 2021). When morphological features were insufficient for reliable identification to the genus or species level, parasites were conservatively classified at higher taxonomic levels (e.g., order or phylum) or recorded as unidentified nematodes, following the diagnostic criteria adopted in the referenced keys.

### 2.6 Data analysis

Data were entered into a spreadsheet and analyzed descriptively. Results were expressed as absolute frequencies and percentages. Overall parasite occurrence was calculated considering the number of samples positive by at least one coproparasitological method divided by the total number of samples examined. Frequencies of the major parasite groups (Nematoda, Trematoda, Cestoda, and Protozoa), as well as single and mixed infections, were calculated among positive samples. Results were additionally described according to the host taxonomic group. The coproparasitological techniques were considered complementary diagnostic approaches, and no inferential comparisons of diagnostic performance among techniques were performed in the present study.

## 3 Results

Of all the samples analyzed, 52.8% (76/144) were positive for endoparasites. Among the positive samples, 80.3% (61/76) harbored nematodes, 10.5% (8/76) trematodes, 13.2% (10/76) cestodes, and 23.7% (18/76) protozoa. In these samples, two or more endoparasites were observed in 26.3% (20/76), characterizing mixed infections, whereas 73.7% (56/76) corresponded to single infections. Descriptively, parasite structures were detected in 55/144 (38.2%) samples using the direct method, 52/144 (36.1%) using spontaneous sedimentation, 47/144 (32.6%) using Willis flotation, and 38/144 (26.4%) using Mini-FLOTAC^®^. These values are presented descriptively and were not used to infer differences in diagnostic performance among methods (**Table 2**).

**Table 2:** Distribution of endoparasites recovered from animals maintained under human care at the Getúlio Vargas Zoobotanical Park, Salvador, BA.

Global distribution (%) of the studied parasites	Mammals, birds, and reptiles
n (%) Positivity	76 (52.8%)
n (%) Negativity	68 (47.2%)
n (%) Total	144 (100.0%)
% Positivity by Direct Method	55 (38.2%)
% Positivity by Willis Flotation	47 (32.6%)
% Positivity by Spontaneous Sedimentation	52 (36.1%)
% Positivity by Mini-FLOTAC®	38 (26.4%)
n (%) Total	144 (100.0)
% Nematoda in positive samples	61 (80.3%)
% Trematoda in positive samples	8 (10.5%)
% Cestoda in positive samples	10 (13.2%)
% Protozoa in positive samples	18 (23.7%)
n (%) Positivity	76 (52.8%)
% Mixed infections in positive samples	20 (26.3%)
% Single infections in positive samples	56 (73.7%)
n (%) Positivity	76 (52.8%)

Source: Authors’ data.

### 3.1 Mammals

Among mammals, 47.8% (44/92) of the samples were positive for endoparasites. Among the positive samples, 75% (33/44) harbored nematodes, 15.9% (7/44) trematodes, 15.9% (7/44) cestodes, and 15.9% (7/44) protozoa. In these samples, mixed infections were observed at 27.3% (12/44), while 72.7% (32/44) corresponded to single infections. Among the 92 samples, parasite structures were detected in 31 (33.7%) by spontaneous sedimentation, 30 (32.6%) by the direct method, 22 (23.9%) by Willis flotation, and 18 (19.6%) by Mini-FLOTAC^®^.

In primates, nematodes such as *Strongyloides* spp. were identified, in addition to representatives of the taxa Ancylostomatidae, Oxyuridae, Ascaridoidea, Strongyloidea, Rhabditida, and unidentified Nematoda eggs. Among cestodes, *Bertiella* spp. was highlighted; among trematodes, *Platynosomum* spp. and unidentified forms; and among protozoa, unsporulated coccidia (**Table 3**; **Figure 2**).

**Table 3:** Results of coproparasitological examinations of mammals from the Getúlio Vargas Zoobotanical Park, Salvador, BA.

Animal species	Results
Black howler monkey (*Alouatta caraya)*	Rhabditida (10 LPG) and *Bertiella* spp.^2^ (20–30 EPG).
Black-faced spider monkey (*Ateles paniscus)*	Ancylostomatidae^2^.
White-cheeked spider monkey (*Ateles marginatus)*	*Strongyloides* spp.^2^ (30 EPG).
Yellow-breasted capuchin (*Sapajus xanthosternos)*	Rhabditida^1^, Ascaridoidea^2^, Ancylostomatidae^2^ (10 EPG) and *Strongyloides* spp.^2^ (10 EPG).
Blond capuchin (*Sapajus flavius)*	Rhabditida^1^, Ancylostomatidae^2^ and *Strongyloides* spp.^2^
Blond titi monkey (*Callicebus barbarabrownae)*	*Bertiella* spp.^2^ (13,750 EPG).
Common squirrel monkey (*Saimiri sciureus)*	Nematoda^2^ (10 EPG), Trematoda^2^, Strongyloidea^2^ (10 EPG), Rhabditida^1^, Ascaridoidea^2^, Ancylostomatidae^2^ (10 EPG), Oxyuridae^2^, *Strongyloides* spp.^2^ (10 EPG) and *Bertiella* spp.^2^ (10 EPG).
Golden-headed lion tamarin (*Leontopithecus chrysomelas)*	Trematoda^2,3^, Strongyloidea^2^, Rhabditida^1^ and *Strongyloides* spp.^2^
Common marmoset (*Callithrix jacchus)*	Strongyloidea^2^, Rhabditida^1^, *Strongyloides* spp.^2^ and *Platynosomum* spp.^2^
Night monkey (*Aotus trivirgatus)*	Strongyloidea^2^, Rhabditida^1^, *Strongyloides* spp.^2^ and unsporulated coccidia^4^.
Crab-eating raccoon (*Procyon cancrivorus)*	Rhabditida^1^ and *Strongyloides* spp.^2^
Lesser grison (*Galictis cuja)*	Rhabditida^1^ and Oxyuridae^2^.
Crab-eating fox (*Cerdocyon thous)*	Oxyuridae^2^.
Brazilian porcupine (*Coendou prehensilis)*	Strongyloidea^2^ (10 EPG), *Strongyloides* spp.^2^, unsporulated coccidia^4^ (3,880 OPG), *Cystoisospora* spp.^4^ (3,020 OPG) and *Entamoeba* spp.^5^
Capybara (*Hydrochoerus hydrochaeris)*	*Strongyloides* spp.^2^
Gray brocket deer (*Subulo gouazoubira)*	Strongyloidea^2^, *Strongyloides* spp.^2^ and *Trichuris* spp.^2^ (10 EPG)
Sambar deer (*Rusa unicolor)*	*Balantioides* spp.^6^
Marsh deer (*Blastocerus dichotomus)*	Unsporulated coccidia^4^.
White-lipped peccary (*Tayassu pecari)*	Nematoda^1^.
Collared peccary (*Pecari tajacu)*	*Balantioides* spp.^5^
Bactrian camel (*Camelus bactrianus)*	Strongyloidea^2^.
Plains zebra (*Equus quagga burchelli)*	Strongyloidea^2^ (100 EPG).
South American tapir (*Tapirus terrestris)*	*Balantioides* spp.^5,6^
White-eared opossum (*Didelphis albiventris)*	Nematoda^1^, Strongyloidea^2^ (270 EPG), Ascaridoidea^2^, Syngamidae^2^ (210 EPG), *Aspidodera* spp.^2^ (140 EPG), *Cruzia* spp.^2^ (560 EPG), unsporulated coccidia^4^ (1,920 OPG) and *Eimeria* spp.^4^
Black-eared opossum (*Didelphis aurita)*	Nematoda^1^, Strongyloidea^2^, Ancylostomatidae^2^, *Cruzia* spp.^2^ and *Trichuris* spp.^2^

^1^ Larvae, ^2^ Eggs, ^3^ Immature stages, ^4^ Oocysts, ^5^ Cysts, ^6^ Trophozoites.

Source: Authors’ data.

**Figure 2:**
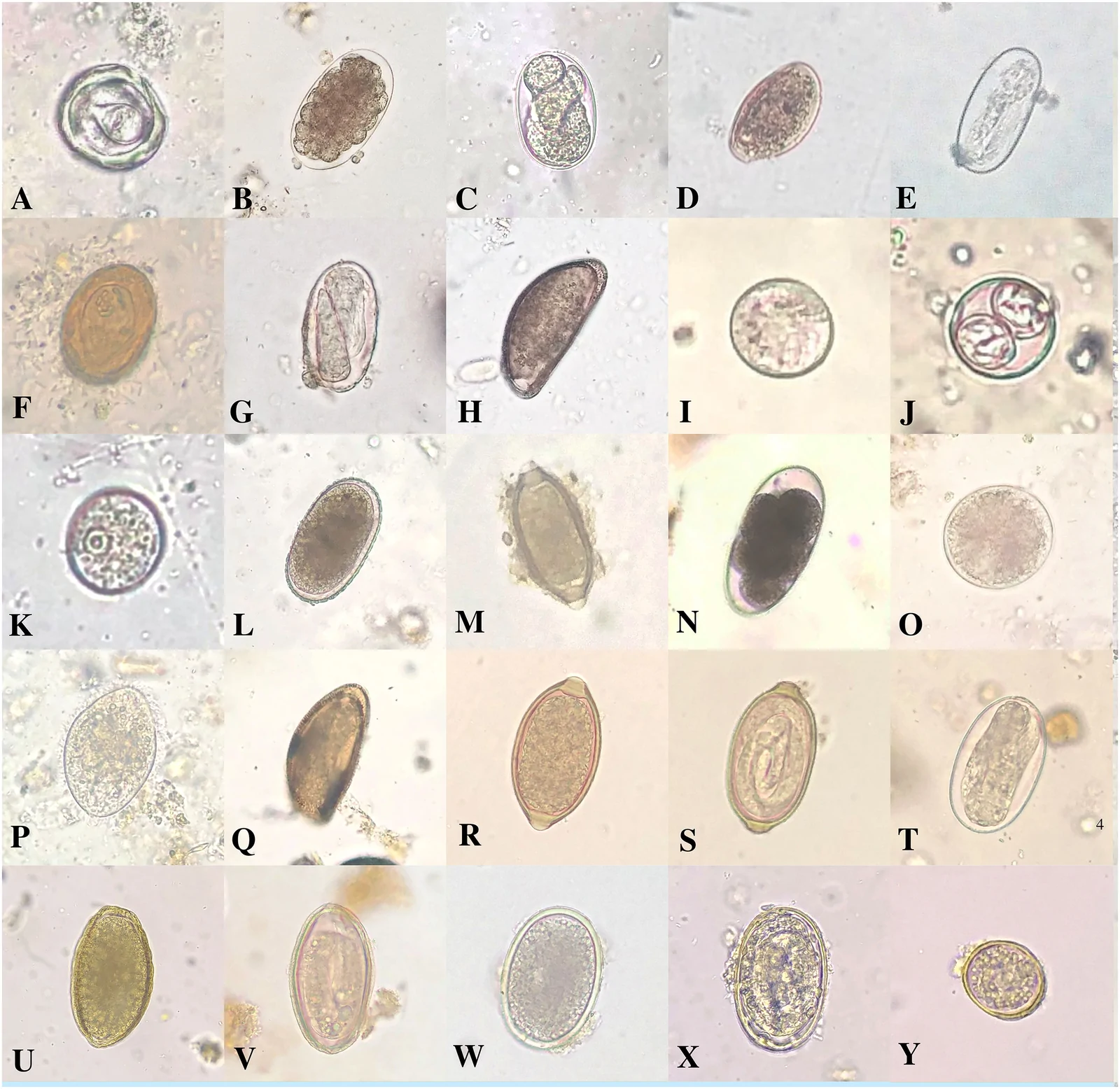
Endoparasites found in mammals from the Getúlio Vargas Zoobotanical Park, Salvador, BA (40× objective). **(A)** – *Bertiella* spp. in black howler monkey (*Alouatta caraya*). **(B)** – Ancylostomatidae in black-faced spider monkey (*Ateles paniscus*). **(C)** – *Strongyloides* spp. in white-cheeked spider monkey (*Ateles marginatus*). **(D)** – Trematoda in common squirrel monkey (*Saimiri sciureus*). **(E)** – Nematoda in *S. sciureus*. **(F)** – *Platynosomum* spp. in common marmoset (*Callithrix jacchus*). **(G)** – *Strongyloides* spp. in crab-eating raccoon (*Procyon cancrivorus*). **(H)** – Oxyuridae in lesser grison (*Galictis cuja*). **(I)** – coccidian in Brazilian porcupine (*Coendou prehensilis*). **(J)** – *Cystoisospora* spp. in *C. prehensilis*. **(K)** – *Entamoeba* spp. in *C. prehensilis*. **(L)** – Strongyloidea in *C. prehensilis*. **(M)** – *Trichuris* spp. in gray brocket deer (*Subulo gouazoubira*). **(N)** – Strongyloidea in *S. gouazoubira*. **(O)** – *Balantioides* spp. (cyst) in collared peccary (*Pecari tajacu*). **(P)** – *Balantioides* spp. (trophozoite) in sambar deer (*Rusa unicolor*). **(Q)** – Oxyuridae in plains zebra (*Equus quagga*). **(R)** – *Trichuris* spp. in black-eared opossum (*Didelphis aurita*). **(S)** – Syngamidae in white-eared opossum (*Didelphis albiventris*). **(T)** – *Strongyloides* spp. in *D. albiventris*. **(U)** – *Cruzia* spp. in *D. aurita*. **(V)** – *Cruzia* spp. (larvated) in *D. albiventris*. **(W)** – *Aspidodera* spp. in *D. albiventris*. **(X)** – *Aspidodera* spp. (larvated) in *D. albiventris*. **(Y)** – coccidian in *D. albiventris*.

In the order Carnivora, *Strongyloides* spp. was observed, as well as representatives of the taxa Oxyuridae and Rhabditida. In rodents, *Strongyloides* spp., nematodes of the order Strongyloidea, as well as unsporulated coccidia, *Cystoisospora* spp., and *Entamoeba* spp. were identified. In artiodactyls, *Strongyloides* spp., *Trichuris* spp., strongyle-type nematodes, *Balantioides* spp., and unsporulated coccidia were detected. In perissodactyls, strongyles and *Balantioides* spp. were also observed. Finally, in didelphids, positivity was observed for the nematodes *Trichuris* spp., *Aspidodera* spp., and *Cruzia* spp., as well as representatives of the taxa Ancylostomatidae, Syngamidae, Ascaridoidea, and Strongyloidea, in addition to unsporulated coccidia and *Eimeria* spp. (**Table 3**; **Figure 2**).

### 3.2 Birds

Among birds, 60.0% (24/40) were positive for endoparasites. Among the positive samples, 87.5% (21/24) harbored nematodes, 4.2% (1/24) trematodes, 12.5% (3/24) cestodes, and 20.8% (5/24) protozoa. In these samples, mixed infections were observed in 25.0% (6/24), while 75.0% (18/24) corresponded to single infections. Among the 40 avian samples examined, parasite structures were detected in 20 (50.0%) by the direct method, 19 (47.5%) by Willis flotation, 16 (40%) by both spontaneous sedimentation and Mini-FLOTAC^®^ (**Table 4**).

**Table 4:** Distribution of endoparasites recovered from animals maintained under human care at the Getúlio Vargas Zoobotanical Park, Salvador, BA, according to host group and coproparasitological technique.

Distribution of endoparasites	Animals		
	Mammals	Birds	Reptiles
n (%) Positivity	44 (47.8%)	24 (60.0%)	08 (66.7%)
n (%) Negativity	48 (52.2%)	16 (40.0%)	04 (33.3%)
n (%) Total	92 (100.0%)	40 (100.0%)	12 (100.0%)
% Positivity by Direct Method	30 (32.6%)	20 (50.0%)	05 (41.7%)
% Positivity by Willis Flotation	22 (23.9%)	19 (47.5%)	06 (50.0%)
% Positivity by Spontaneous Sedimentation	31 (33.7%)	16 (40.0%)	05 (41.7%)
% Positivity by Mini-FLOTAC®	18 (19.6%)	16 (40.0%)	04 (33.3%)
n (%) Total	92 (100.0%)	40 (100.0%)	12 (100.0)
% Nematoda in positive samples	33 (75.0%)	21 (87.5%)	07 (87.5%)
% Trematoda in positive samples	07 (15.9%)	01 (4.2%)	–
% Cestoda in positive samples	07 (15.9%)	03 (12.5%)	–
% Protozoa in positive samples	7 (15.9%)	05 (20.8%)	06 (75.0%)
n (%) Positivity	44 (100.0%)	24 (100.0%)	08 (100.0%)
% Mixed infections in positive samples	12 (27.3%)	6 (25.0%)	02 (25.0%)
% Single infections in positive samples	32 (72.7%)	18 (75.0%)	06 (75.0%)
n (%) Positivity	44 (100.0)	24 (100.0)	08 (100.0)

Source: Authors’ data.

Psittacines were positive for *Capillaria* spp., Ascaridoidea, Strongyloidea, and eggs of Nematoda and Cestoda. In passerines, *Isospora* spp., unsporulated coccidia, and eggs of Nematoda and Cestoda were identified. In *Butorides striata*, strongyle-type and Nematoda eggs were recovered. In *Rhamphastos toco* and *Cariama cristata*, nematode eggs were observed. In *Pavo cristatus*, eggs of *Capillaria* spp. were identified, while in *Crax fasciolata*, eggs of *Strongyloides* spp., Nematoda, and unsporulated coccidia were found (**Table 5**; **Figure 3**).

**Table 5:** Results of coproparasitological examinations of birds from the Getúlio Vargas Zoobotanical Park, Salvador, BA.

Animal species	Results
Macaws (*Ara* spp.)	Cestoda^2^, Nematoda^1^ (10 LPG), Strongyloidea^2^, Ascaridoidea^3^ (50 EPG) and *Capillaria* spp.^2^ (10 EPG).
Hyacinth macaw (*Anodorhynchus hyacinthinus)*	Nematoda^1^ and *Capillaria* spp.^2^ (300 EPG).
Blue-fronted amazon (*Amazona aestiva)*	Ascaridoidea^2^ (60–460 EPG) and *Capillaria* spp.^2^
Red-browed amazon (*Amazona rhodocorytha)*	Ascaridoidea^2^ (2,100-3,700 EPG) and *Capillaria* spp.^2^ (50 EPG).
Peach-fronted parakeet (*Eupsittula aurea)*	*Capillaria* spp.^2^ (20 EPG).
Golden-capped parakeet (*Aratinga auricapillus)*	Ascaridoidea^2^ (40 EPG)
Red-shouldered macaw (*Diopsittaca nobilis)*	Nematoda^1^, Ascaridoidea^2^ (50 EPG) and *Capillaria* spp.^2^ (20 EPG).
Red-fan parrot (*Deroptyus accipitrinus)*	Nematoda^1^, Ascaridoidea^2^ (50 EPG) and *Capillaria* spp.^2^ (20 EPG).
Rufous-bellied thrush (*Turdus rufiventris)*	Nematoda^1^, Cestoda^2^ (150 EPG), unsporulated coccidia^4^ (10-1,780 OPG) and *Isospora* spp.^4^ (1,280 OPG).
Sayaca tanager (*Thraupis sayaca)*	Unsporulated coccidia^4^ (20,400-80,304 OPG) and *Isospora* spp.^4^ (4,320-7,080 OPG).
Striated heron (*Butorides striata)*	Trematoda^2^ and Strongyloidea^2^.
Toco toucan (*Rhamphastos toco*)	Nematoda^1^.
Indian peafowl (*Pavo cristatus)*	*Capillaria* spp.^2^
Bare-faced curassow (*Crax fasciolata)*	Nematoda^1^, *Strongyloides* spp.^2^ and unsporulated coccidia^4^.
Red-legged seriema (*Cariama cristata)*	Nematoda^1^.

^1^ Larvae, ^2^ Eggs, ^3^ Immature stages, ^4^ Oocysts, ^5^ Cysts, ^6^ Trophozoites.

Source: Authors’ data.

**Figure 3:**
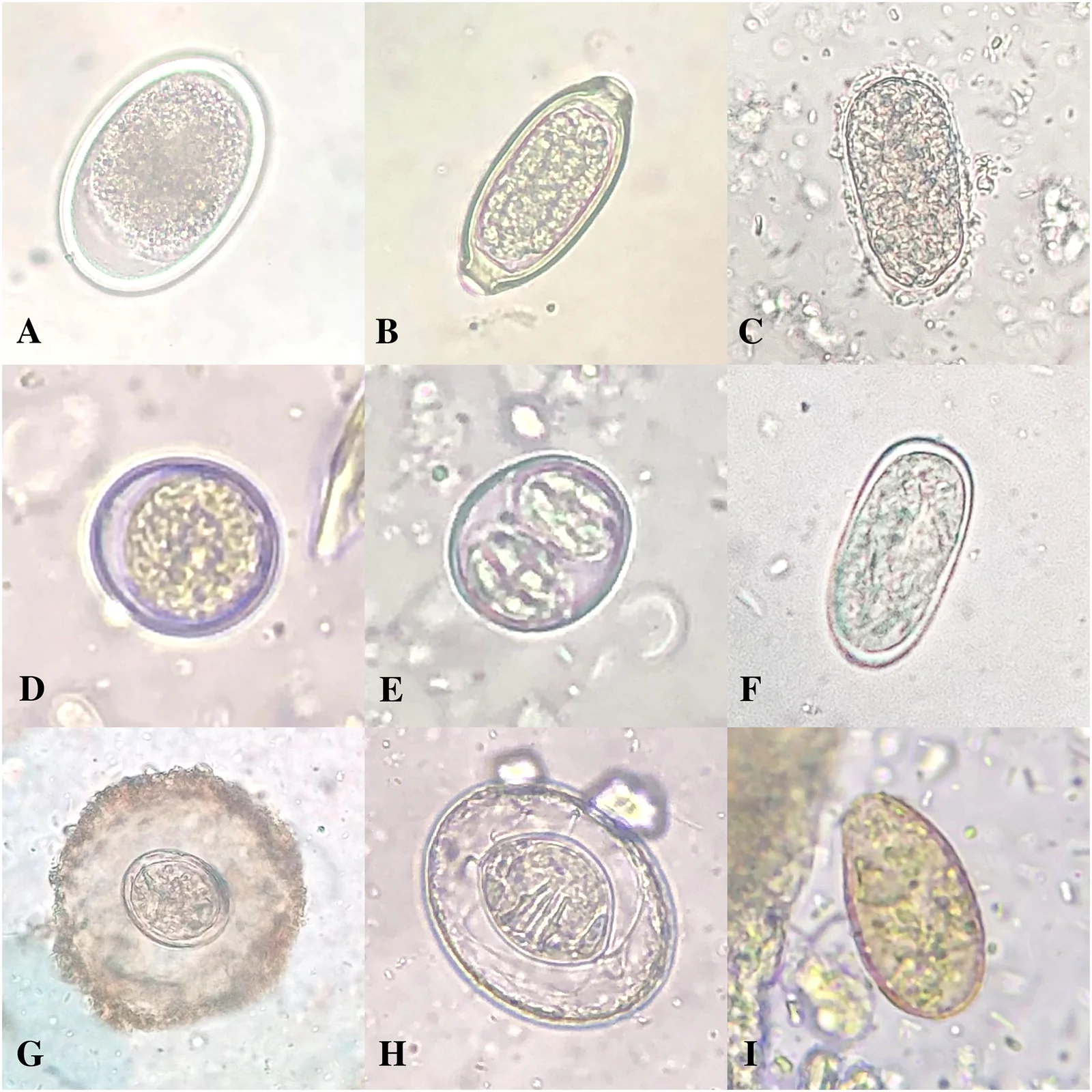
Endoparasites found in birds from the Getúlio Vargas Zoobotanical Park, Salvador, BA (40× objective). **(A)** – Ascaridoidea in blue-fronted amazon (*Amazona aestiva*). **(B)** – *Capillaria* spp. in hyacinth macaw (*Anodorhynchus hyacinthinus*). **(C)** – Strongyloidea in macaws (*Ara* spp.). **(D)** – coccidian in rufous-bellied thrush (*Turdus rufiventris*). **(E)** – *Isospora* spp. in sayaca tanager (*Thraupis sayaca*). **(F)** – Strongyloidea in striated heron (*Butorides striata*). **(G)** – Cestoda in macaws. **(H)** – Cestoda in rufous-bellied thrush (*T. rufiventris*). **(I)** – Trematoda in *B. striata*.

### 3.3 Reptiles

Among reptiles, 66.7% (8/12) of the samples were positive for endoparasites. Among the positive samples, 87.5% (7/8) harbored nematodes and 75.0% (6/8) protozoa. In these samples, mixed infections were observed in 25.0% (2/8), while 75.0% (6/8) corresponded to single infections. Among the 12 reptile samples examined, parasite structures were detected in 6 (50.0%) by Willis flotation, 5 (41.7%) by the direct method, 5 (41.7%) by spontaneous sedimentation, and 4 (33.3%) by Mini-FLOTAC^®^ (**Table 4**).

In snakes, coccidia, rhabditiform larvae, and eggs of the taxa Strongyloidea were identified. In *Iguana iguana*, coccidia and eggs of Oxyuridae were observed. In *Chelonoidis carbonaria*, cysts and trophozoites of *Balantioides* spp. and *Nyctotherus* spp. were recovered, in addition to coccidia, strongyle-type eggs, and larvae of Atractidae (**Table 6**; **Figure 4**).

**Table 6:** Results of coproparasitological examinations of reptiles from the Getúlio Vargas Zoobotanical Park, Salvador, BA.

Animal species	Results
Rainbow boa (*Epicrates cenchria x assisi*)	Unsporulated coccidia^4^ (10 OPG).
Burmese python (*Python molurus)*	Rhabditida^1^.
Bushmaster (*Lachesis muta)*	Rhabditida^1^, Strongyloidea^2^ (10 EPG) and unsporulated coccidia^4^ (270 OPG).
Green iguana (*Iguana iguana)*	Oxyuridae^2^ and unsporulated coccidia^4^.
Red-footed tortoise (*Chelonoidis carbonaria)*	Strongyloidea^2^, Atractidae^1^ (10 LPG), unsporulated coccidia^4^, *Nyctotherus* spp.^5,6^ and *Balantioides* spp. ^5,6^.

^1^ Larvae, ^2^ Eggs, ^3^ Immature stages, ^4^ Oocysts, ^5^ Cysts, ^6^ Trophozoites.

Source: Authors’ data.

**Figure 4:**
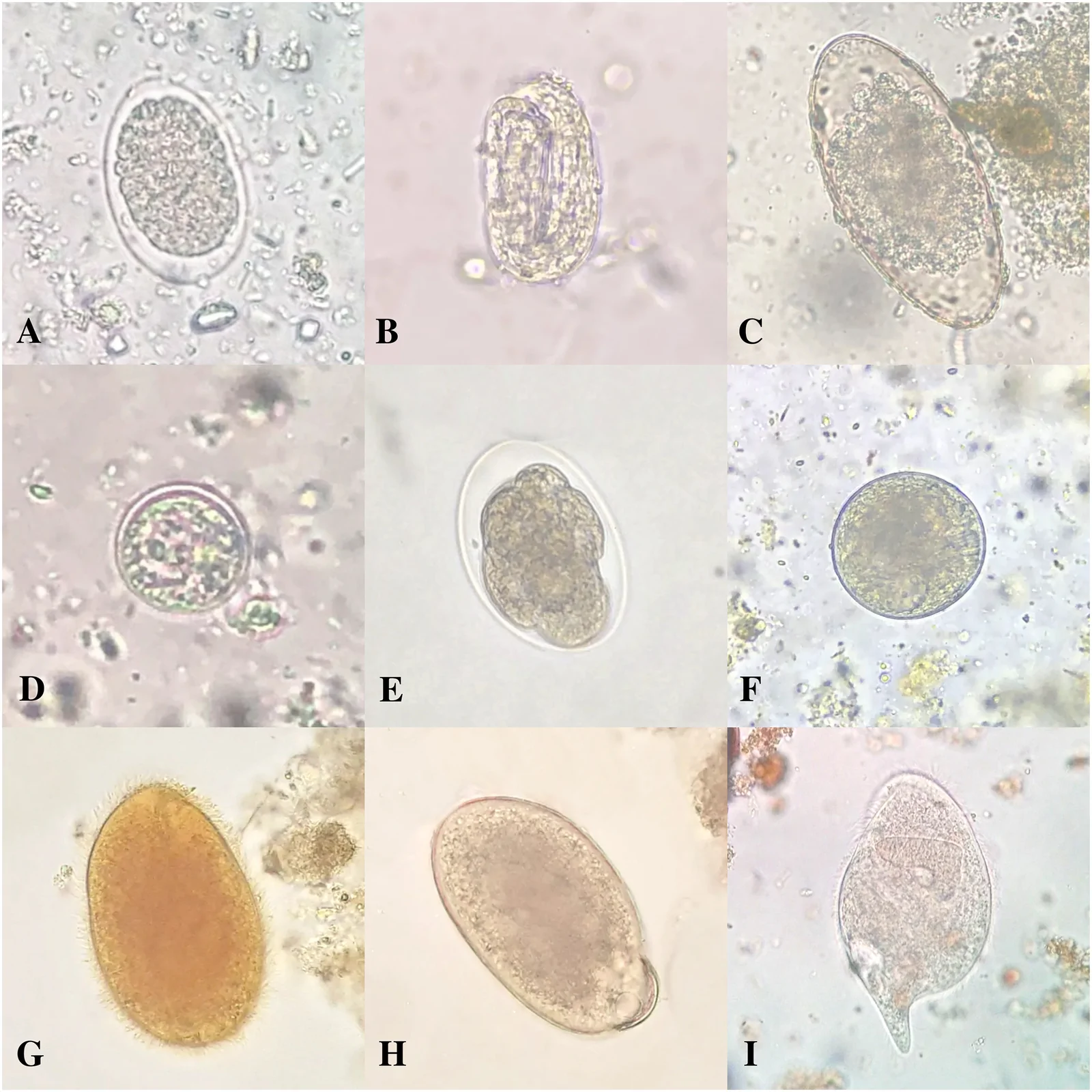
Endoparasites found in reptiles (40× objective). **(A)** – *Strongyloidea* in bushmaster (*Lachesis muta*). **(B)** – Strongyloidea in *L. muta*). **(C)** – Oxyuridae in green iguana (*Iguana iguana*). D – Coccidian in rainbow boa (*Epicrates cenchria × assisi*). **(E)** – *Strongyloidea* in red-footed tortoise (*Chelonoidis carbonaria*). **(F, G)** – *Balantioides* spp. (**F** – cyst; **G** – trophozoite) in C. *carbonaria*. **(H, I)** – *Nyctotherus* spp. (**H** – cyst; **I** – trophozoite) in *C. carbonaria*.

## 4 Discussion

Several studies conducted in zoological settings have evaluated the occurrence of endoparasites in mammals, birds, and reptiles, generally reporting higher frequencies in birds and mammals compared to reptiles (Batista et al., 2021; Santos et al., 2022; Mewius et al., 2021; Silva et al., 2022). However, during the single sampling period evaluated in the present study, reptiles showed the highest positivity rate (66.7%), followed by birds (60.0%) and mammals (47.8%), indicating an inversion of the commonly described pattern. This variation may be related to the sampled species and the characteristics of parasitism in reptiles, which is often asymptomatic or oligosymptomatic, making clinical identification more difficult (Batista et al., 2021).

Regarding parasitic groups, there was a predominance of nematodes (80.3%), followed by protozoa (23.7%), cestodes (13.2%), and trematodes (10.5%), values similar to those reported in the literature (Batista et al., 2021). The higher frequency of nematodes and protozoa may be explained by their direct life cycle, which does not require intermediate hosts, favoring transmission in captive environments (Barbosa et al., 2019). In addition, as reported in other studies, single infections predominated over mixed infections. This pattern was also observed by Fagiolini et al. (2010); Snak et al. (2014), and Capasso et al. (2019), and is consistent with the present findings.

In mammals, the frequency of endoparasitism observed was consistent with the literature, which reports positivity rates between 40% and 60%, with predominance of nematodes and single infections (Fagiolini et al., 2010; Snak et al., 2014; Capasso et al., 2019). In birds, the findings also followed the described pattern, with rates between 30% and 60% and a higher occurrence of nematodes, followed by protozoa (Melo et al., 2013; Snak et al., 2014; Hofstatter and Guaraldo, 2015; Manjunatha et al., 2023). In contrast, reptiles showed higher parasitism rates than those reported in previous studies, which generally range from 5% to 55% (Mewius et al., 2021; Santos et al., 2022; Silva et al., 2022). Unlike some studies that report a predominance of protozoa in these animals, a higher occurrence of nematodes was observed (Fernando and Udagama-Randeniya, 2009; Santos et al., 2022).

The use of complementary coproparasitological methods, including direct examination, spontaneous sedimentation, Willis flotation, and Mini-FLOTAC^®^, allowed the recovery of a broad diversity of parasitic structures. Because these approaches differ in their ability to recover eggs, larvae, cysts, and oocysts with distinct physical and biological characteristics, their complementary use may broaden the spectrum of detectable parasites. In the present study, the techniques were therefore used as complementary diagnostic approaches rather than as competing tests, and differences in their diagnostic performance were not formally evaluated (Fagiolini et al., 2010; Batista et al., 2021; Cringoli et al., 2010, 2013, 2017). A limitation related to the Mini-FLOTAC^®^ procedure is that the dilution used in the present study followed the manufacturer’s recommendation for dogs and cats. Because the study included mammals, birds, and reptiles, this dilution may not be equally optimized or validated for all host groups evaluated. Therefore, the Mini-FLOTAC^®^ results should be interpreted with caution, particularly when comparisons across taxonomic groups are considered.

Flotation techniques, such as Willis and Mini-FLOTAC^®^, are generally more effective for recovering light eggs and oocysts because these structures readily float in solutions with high specific gravity (Ballweber et al., 2014; Maurelli et al., 2014). Conversely, sedimentation techniques are more suitable for detecting heavier eggs, including those of trematodes, which may not efficiently float in saturated solutions (Becker et al., 2016). The direct method, although less sensitive in low-intensity infections, enables the rapid detection of a wide range of parasitic forms, including larvae, protozoan stages, and eggs present in fresh samples (Zajac and Conboy, 2021). Therefore, the diagnostic performance of each technique depends not only on the host species but also on the characteristics of the parasitic structures being examined, reinforcing the importance of combining complementary methods for a more comprehensive parasitological diagnosis (Verocai et al., 2020; Lignon et al., 2025).

The presence of endoparasites with zoonotic potential is also noteworthy, such as *Balantioides* spp., *Strongyloides* spp., *Trichuris* spp., and *Platynosomum* spp., as well as representatives of Strongyloidea, Ascaridoidea, Ancylostomatidae, and Oxyuridae (Fagiolini et al., 2010; Lima et al., 2017; Batista et al., 2021; Silva et al., 2022). Among the main novel findings of this study are the first records of this parasite taxon of *Strongyloides* spp. in white-cheeked spider monkey (*Ateles marginatus*) and golden-headed lion tamarin (*Leontopithecus chrysomelas*), *Bertiella* spp. In blond titi monkey (*Callicebus barbarabrownae*) and common squirrel monkey (*Saimiri sciureus*), and *Capillaria* spp. in red-browed amazon (*Amazona rhodocorytha*), peach-fronted parakeet (*Eupsittula aurea*), and red-shouldered macaw (*Diopsittaca nobilis*). Additionally, *Cystoisospora* spp. and *Entamoeba* spp. were identified in brazilian porcupine (*Coendou prehensilis*), expanding the knowledge of the parasitic fauna of these species.

This study has some limitations. Fecal samples were collected as pooled samples from enclosure floors; therefore, the results should be interpreted at the sample or enclosure level rather than as individual frequency. Because several individuals could contribute to the same composite fecal sample, samples obtained from multi-individual enclosures cannot be considered statistically independent observations at the individual level. Consequently, the enclosure constituted the sampling unit, and the results should be interpreted only at the enclosure/sample level rather than as estimates of individual prevalence. Another limitation of this study is its cross-sectional design. Fecal samples were collected during a single sampling period (December 2021), providing only a point-in-time snapshot of parasite positivity in the evaluated enclosures. Consequently, seasonal fluctuations in parasite occurrence, shedding patterns, and environmental conditions could not be assessed. Longitudinal studies including repeated sampling across different seasons are needed to better characterize temporal variation in parasite circulation.

In addition, environmental contamination cannot be completely ruled out, despite the precautions adopted during collection. The use of formalin-preserved feces and the absence of molecular confirmation also limited parasite identification to morphological categories in some cases (Hass et al., 2024). Another limitation of this study is the absence of calibrated morphometric measurements and scale bars in the photomicrographs. Owing to limitations of the laboratory equipment available during image acquisition, it was not possible to obtain standardized measurements of the parasitic structures. Nevertheless, microscopic identification was performed by experienced parasitologists based on established morphological criteria, considering the shape, internal characteristics, and the approximate dimensions of eggs, oocysts, cysts, trophozoites, and larvae according to the objective magnification used during examination. Although morphometric data would provide additional support for the identification and facilitate independent verification, the combination of these morphological characteristics allowed reliable classification of the parasitic structures observed.

This study highlights the diversity of endoparasites in wild animals maintained in captivity and emphasizes the importance of coproparasitological diagnosis in this context. Parasitological monitoring is essential for maintaining animal health, supporting appropriate management practices, and reducing the risk of parasite transmission, including zoonotic infections. These findings reinforce the importance of continued research and surveillance of parasitic infections in captive wild animals.

## Data Availability

The original contributions presented in the study are included in the article/supplementary material. Further inquiries can be directed to the corresponding author.
